# Unobtrusive Nighttime Movement Monitoring to Support Nursing Home Continence Care: Algorithm Development and Validation Study

**DOI:** 10.2196/58094

**Published:** 2024-12-24

**Authors:** Hannelore Strauven, Chunzhuo Wang, Hans Hallez, Vero Vanden Abeele, Bart Vanrumste

**Affiliations:** 1 e-Media Research Lab/STADIUS, Department of Electrical Engineering, KU Leuven, Andreas Vesaliusstraat 13, Leuven, 3000, Belgium, +32 16377662; 2 Research Group M-Group/DistriNet, Department of Computer Science, KU Leuven, Brugge, Belgium; 3 e-Media Research Lab/Human-Computer Interaction, Department of Computer Science, KU Leuven, Leuven, Belgium

**Keywords:** nursing home, agitation, incontinence, accelerometer, unobtrusive, enuresis, sensor technology

## Abstract

**Background:**

The rising prevalence of urinary incontinence (UI) among older adults, particularly those living in nursing homes (NHs), underscores the need for innovative continence care solutions. The implementation of an unobtrusive sensor system may support nighttime monitoring of NH residents’ movements and, more specifically, the agitation possibly associated with voiding events.

**Objective:**

This study aims to explore the application of an unobtrusive sensor system to monitor nighttime movement, integrated into a care bed with accelerometer sensors connected to a pressure-redistributing care mattress.

**Methods:**

A total of 6 participants followed a 7-step protocol. The obtained dataset was segmented into 20-second windows with a 50% overlap. Each window was labeled with 1 of the 4 chosen activity classes: in bed, agitation, turn, and out of bed. A total of 1416 features were selected and analyzed with an XGBoost algorithm. At last, the model was validated using leave one subject out cross-validation (LOSOCV).

**Results:**

The trained model attained a trustworthy overall *F*_1_-score of 79.56% for all classes and, more specifically, an *F*_1_-score of 79.67% for the class “Agitation.”

**Conclusions:**

The results from this study provide promising insights in unobtrusive nighttime movement monitoring. The study underscores the potential to enhance the quality of care for NH residents through a machine learning model based on data from accelerometers connected to a viscoelastic care mattress, thereby driving progress in the field of continence care and artificial intelligence–supported health care for older adults.

## Introduction

### Background

With the increase in life expectancy, there is a corresponding rise in the prevalence of urinary incontinence (UI), a common health problem among older adults [1]. Studies have indicated that UI affects over 50% of older adults residing in nursing homes (NHs) [2-4]. Current care practices for managing UI in NHs involve incontinence wear (ie, disposable absorbent products), with or without scheduled toilet visits (voiding) [5]. Unfortunately, these practices often lead to redundant checks and delayed interventions, thereby triggering undesirable consequences, such as disturbed sleep patterns [6]. Despite its significant impact for residents’ health and overall quality of life, along with the increased burden it places on care personnel and noteworthy financial implications, UI remains underdiagnosed and its management underreported [7].

In a recent scoping review conducted by Omotunde and Wagg [8], authors found encouraging outcomes regarding technology-driven continence care with a majority of solutions incorporating sensor technology integrated into body-worn disposable absorbent products (colloquially referred to as “smart diapers”). These smart diapers offer feedback regarding saturation levels and are capable of identifying instances of leakage [9]. Complementary software apps are used to collate these data and generate insightful information and reminders intended for care personnel.

The introduction of technology-based continence care within NHs, facilitated by the use of smart diapers, holds the potential to monitor voiding processes and allows for timely product changes [10]. By embracing such advancements, NHs could potentially improve the quality of care delivered to residents with UI.

The technology, however, comes with significant expenses, with an initial installation and training cost up to US $3300, combined with an additional charge of up to US $3.50 per diaper for the incontinence wear [11]. Consequently, smart diapers are mainly used for a short period of time (ie, 3 days) to establish a personalized continence care plan of the resident [10,12]. Furthermore, the use of smart diapers is intrinsically linked to the use of continence wear, limiting the ability to monitor UI patterns exclusively during periods when these absorbent products are worn. This contrasts with the research findings of Ostaszkiewicz et al [13], which emphasized the urgent need for independent resources, for example, technology, to inform decision-making regarding continence wear.

Moreover, previous research [14] underlined the significance of developing technology solutions that exhibit sensitivity toward issues of intimacy, stigma, and taboo inherent in continence care to preserve the NH residents’ dignity and overall quality of life. In light of this perspective, the design of a monitoring system for NHs should prioritize unobtrusiveness, discreteness, and compatibility with appropriate care equipment.

Prior research has explored monitoring nighttime movement and identifying sleep-related disorders or sleep stages via the use of unobtrusive sensor systems equipped with accelerometer or pressure sensors, connected to beds [15-18]. However, only a limited number of researchers have directed the focus of nighttime movement monitoring with accelerometer sensors connected to the bed toward the exploration of detecting nighttime movement to support NH continence care. These few studies [19-21] are listed in Table 1 and are further detailed in the section *Prior Work*.

**Table 1.** Overview of unobtrusive accelerometer sensor systems evaluated to monitor nighttime agitation with a relation to continence care, summarizing the number of participants (p) and location of the study setup, the sensor position on the mattress, and the algorithm deployed for data analysis.

Authors and study	Study setup	Sensor position (mattress)	Algorithm
Gong et al [19]	12 p at home	Top and bottom + wristbands	Cole‘s actigraphy [22] and STFT^a^ [23]
T’Jonck et al [20]	4 p at home	Top	CNN^b^
T’Jonck et al [21]	1 p in lab	Bottom	FFT^c^ and CNN

a STFT: short-time Fourier transform.

b CNN: convolutional neural network.

c FFT: fast Fourier transform.

### Prior Work

Gong et al [19] monitored nighttime movement and incontinence in patients with Alzheimer disease. Their study encompassed 12 participants in a home environment. Wetness events were monitored via the wireless bed-wetting alarm system DryBuddy [24]. The system used two triaxial accelerometer sensors, positioned on the upper and lower sides of the mattress. They applied Cole’s actigraphy algorithm [22] on the sensor data to estimate wake and sleep periods. Two additional accelerometer sensors [25] were strapped to both wrists of participants to monitor hand movements. For the nighttime sleep agitation assessment, they calculated a short-time Fourier transform [23], based on a combined dataset from the bed sensors data and wrists’ nodes data, to indicate agitation.

The authors established that almost half (49%) of the sleep agitation events occurred before a voiding event, supporting the observation that a need to void can trigger agitation. However, authors did not provide evaluation metrics for the used algorithm, nor differentiated multiple nighttime activities.

In another study, T’Jonck et al [20] deployed a smartphone-integrated triaxial accelerometer, which was placed in 4 different positions on the mattress. Their study encompassed 4 participants within a home setting, using a convolutional neural network (CNN) approach for nighttime activity tracking (ie, none, sit down, lay down, sit up, and stand up). When including all sensor positions in the model, an accuracy (ie, the ratio of correct predictions to the total number of predictions [26]) of 92% was reached. Unfortunately, authors did not further elaborate on these different positions.

In a subsequent study of T’Jonck et al [21], the smartphone was substituted with an triaxial accelerometer sensor, positioned on the bottom surface of the mattress. This evaluation was conducted with one participant within a laboratory environment, and activity tracking (ie, none, in bed, out of bed, changing position, and agitation) was accomplished via a fast Fourier transform (FFT) model in addition to a CNN-based model. For the model that combines FFT and CNN, an accuracy of 88.96% was achieved, showing the applicability of unobtrusive monitoring of nighttime movement via accelerometer sensors.

Both studies yield outcomes that suggest promise for the detection of agitation and, hence, monitoring nighttime movement. However, Gong et al’s [19] system design involved the use of nodes strapped on the participants’ wrists, which could be perceived as obtrusive. Furthermore, their study did not include classification metrics to evaluate the used algorithm, hindering a meaningful comparison with alternative system designs.

Conversely, T’Jonck et al [20,21] prioritize an unobtrusive system design in their research, incorporating thorough evaluation metrics for their developed algorithms. Consequently, in their subsequent study, they substitute the smartphone app with a mattress-attached sensor. However, this modified setup is only validated using data from a single participant.

Both studies were conducted in a home environment or explicitly specified the use of a standard bed and mattress, without considering the pressure-redistributing features of care mattresses for care beds. Such a care mattress is composed of a temperature-sensitive cell structure that softens by the heat from an individual’s body and molds around the body to distribute pressure efficiently [27]. This means that the individual’s weight can spread over a much wider area compared with a conventional mattress. Such a care mattress is frequently used in NHs, as it reduces the risk of pressure ulcers and, thus, is recommended for use among individuals at high risk of developing pressure ulcers [28-30].

### Goal of This Study

In this study, we extend the investigation of unobtrusive monitoring with accelerometer sensors positioned on the bottom surface of a pressure-redistributing care mattress. This exploration aims to monitor nighttime movement and detect large body movements, a symptom of nocturnal agitation [31], simulated by 6 adult participants in an experimental setup. Notably, our approach incorporates a care bed with a viscoelastic mattress as used in NH settings, for the purpose of tracking 4 activities: in bed, turn, agitation, and out of bed. Through this methodology, we endeavor to enhance the understanding of the potential of using artificial intelligence tools in advancing the field of NH continence care.

## Methods

### Movement Monitoring System

The movement of the participants was monitored using the Byteflies Kit [32], a medically certified motion monitoring device, with sensor dots. A single sensor dot can record triaxial accelerometer and triaxial gyroscope signals, sampled at 100 Hz. Sensor dots can last 24 hours and are charged via a docking station.

**Figure 1.**
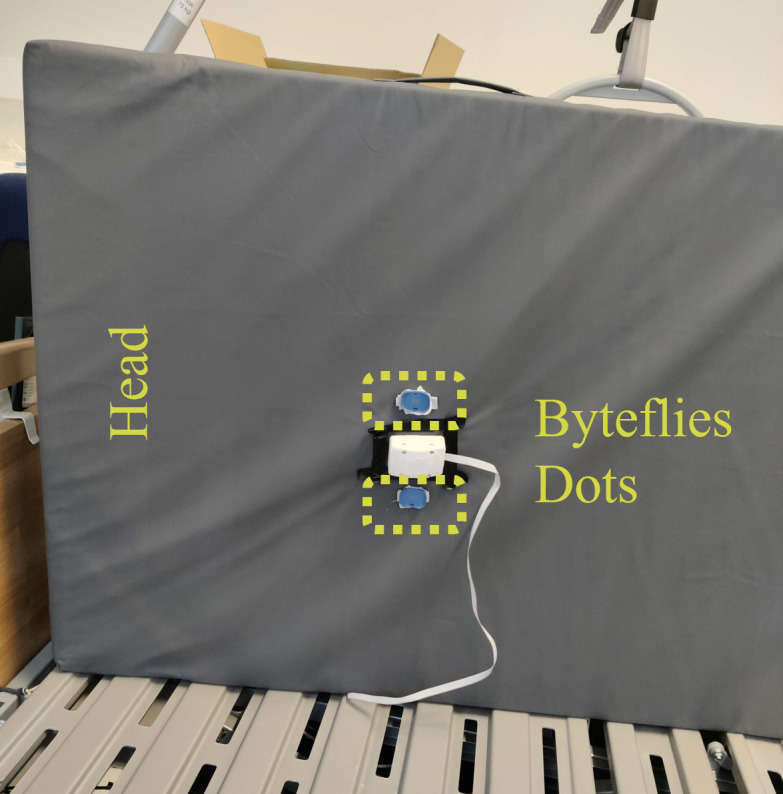
Photograph of the movement monitoring system attached to the bottom side of the mattress on a nursing home care bed. The position of the two Byteflies sensor dots is circled by a dotted line, with dot 1 positioned on top and dot 2 on the bottom.

In this measurement, the researchers opted to attach two sensor dots to the bottom side of the mattress on an NH care bed (Figure 1) on the left and right side. The placement of the dots on the mattress aligned with the positioning in a previous study [33]. If a participant lay down on the care bed, the sensor dots were located beneath their back.

The pressure-redistributing care mattress is a Tempur-Med viscoelastic mattress with a width of 14 cm, as commonly used in NHs to reduce pressure ulcers [27].

### Recruitment

The data acquisition was carried out during the COVID-19 pandemic, from October 2020 until December 2020. NH residents are a frail population and were greatly affected by the adverse health effects of the pandemic. Therefore, we recruited university colleagues who were allowed to travel to campus. Ethical approval to conduct the research was obtained from the KU Leuven Social and Societal Ethics Committee with protocol number G-2020‐2214. Safety measures as mandated by the national government were applied at all times. Inclusion criteria for participants were individuals 18 years or older, living in Belgium, and being able to participate independently, understanding the purpose and involvement and providing consent. In total, 6 colleagues volunteered their time with a mean age of 29 (SD=4) years, a mean height of 177(SD=9) cm, and a mean weight of 74(SD=20) kg. Among the participants, 2 were female, and 4 were male.

### Measurement Protocol

**Figure 2.**
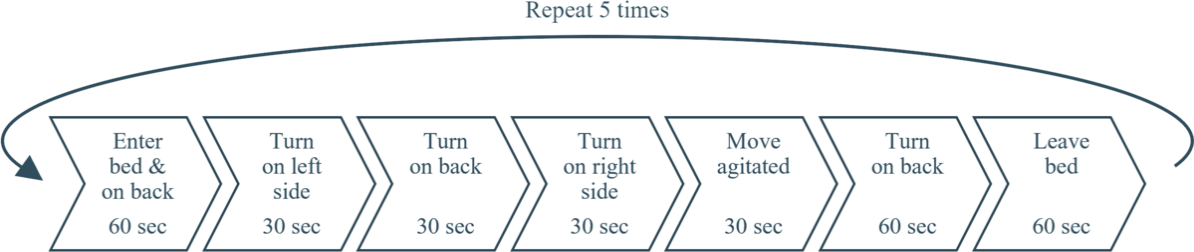
Illustration of the measurement protocol followed by the participants to monitor their movement.

The participants were instructed to follow a 7-step protocol outlined in Figure 2 to simulate nighttime movement, including (nocturnal) agitation. To start with step 1, the participants entered the care bed on their back, lying down for 60 seconds. Subsequently, for step 2, they turned onto their left side and waited for 30 seconds. This sequence continued with step 3, involving a return to their back and a 30-second wait, followed by step 4, requiring a turn to their right side with another 30-second interval. In step 5, participants engaged in large body movements for 30 seconds, with the execution left to the participants’ interpretation, because the authors could not find a standardized definition or duration for nocturnal agitation in older adults correlated to incontinence. Step 5 was succeeded by step 6, involving lying on their back for 60 seconds. Finally, step 7 required participants to leave the bed for 60 seconds before repeating the entire protocol. Each participant completed the protocol 5 times. A Garmin Venue SQ smartwatch [34] guided the participants through the protocol, providing vibration notifications to prompt transitions between steps.

### Data Collection and Analysis

First, relevant settings and hyperparameters to start the data analysis and training process were tuned on the basis of the obtained results. After an exploration to identify the optimal settings, the following configurations were selected.

#### Signal Preprocessing and Annotation

**Figure 3.**
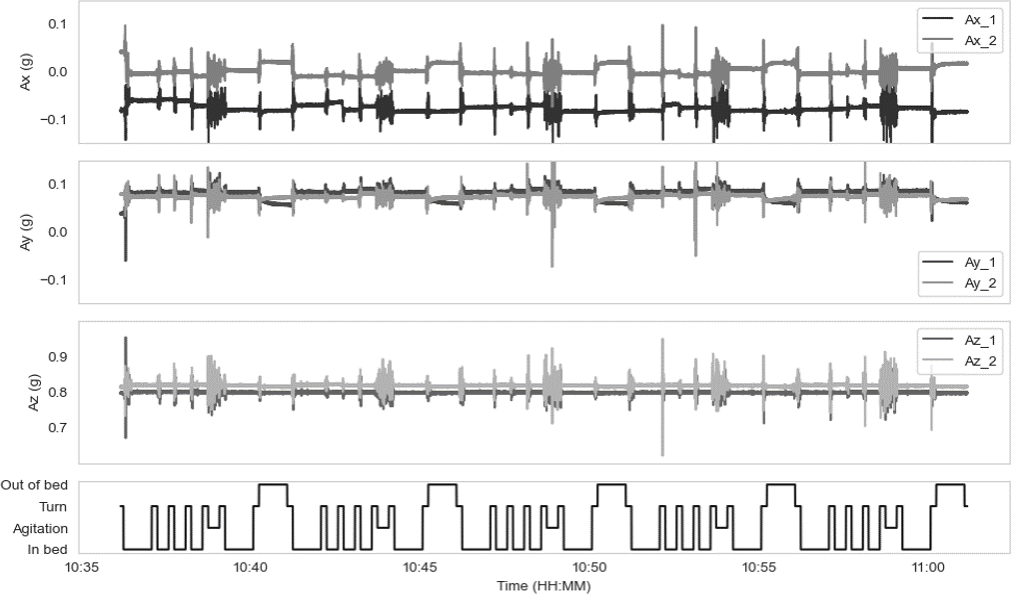
The unfiltered triaxial acceleration (Ax, Ay, and Az) in gravity (g) in time of two Byteflies dots for participant 2 for a complete measurement protocol. Beneath this sensor data, the signal is categorized into 4 activity classes.

Figure 3 displays the unfiltered triaxial accelerometer data gathered from the 2 Byteflies dots for participant 2. These data were annotated based on the protocol’s time interval. In this preprocessing, the data were also scaled to the unit variance and band-pass filtered. Starting from the filter settings initially outlined by Razjouyan et al [35] and further fine-tuned for our dataset, a fourth-order Butterworth filter was applied with cutoff frequencies at 2 Hz and 10 Hz. At last, the data were categorized into 4 activity classes that are relevant for monitoring with regard to NH (continence) care management:

1. In bed: The bed is occupied, and the participant lies either on their back (steps 1, 3, and 6), left side (step 2), or right side (step 4). For NH residents, activity within this class is considered regular nighttime behavior.
2. Agitation: The bed is occupied, and the participant acts agitated by moving their arms and legs (step 5). For NH residents who need continence care, this agitated movement can be triggered by a voiding event.
3. Turn: The bed is occupied, and the participant transitions from the current step in the protocol to the following step (eg, from turning on the left side in step 2 to turning on the back in step 3). On each occasion in the measurement protocol, the last 5 seconds of the current step and the first 5 seconds of the following step are categorized as a turn. For NH residents, turning is considered to be an effective way of preventing pressure ulcers [36].
4. Out of bed (unoccupied): The bed is unoccupied, as the participant left the bed. This is regular daytime behavior for NH residents.

#### Sliding Window

A sliding window was applied on the dataset and the selected window size was 20 seconds with 50% overlapping for a sampling frequency of 100 Hz. If there were multiple classes present within one window, the window was annotated as the majority class.

#### Feature Extraction and Selection

To extract features from the segmented time series dataset, the Time Series Feature Extraction Library Python package was selected [37]. Time Series Feature Extraction Library is an automated process of feature extraction, designed to accelerate the time consuming and complex exploratory analysis of multidimensional time series. The library computes over 60 different parameters across temporal, statistical, and spectral domains. Out of the computed set of 4668 features, a refined subset of 1416 features was derived after eliminating correlated and zero-variance features. Subsequently, this selected subset was scaled to unit variance.

#### Machine Learning Algorithm

On the basis of the selected features and obtained results in the training process, the scalable end-to-end tree extreme gradient boosting system, XGBoost, was used to train the model for the classification task at hand [38]. It is an open-source Python package that implements gradient boosting and tree learning paralleling, effective in applications with limited data and in human activity recognition, including older adults [38-41]. The hyperparameters of the XGBoost algorithm were optimally adapted to obtain a robust and accurate model. SHAP (Shapley additive explanations) TreeExplainer was used as the explanation method for the model’s output, providing fast local explanations with guaranteed consistency [42].

#### Model Training and Evaluation

To evaluate the model, leave one subject out cross-validation (LOSOCV) was used. This statistical technique divides the original dataset into a training and validation set, alternating between them in successive rounds and ensuring each data point undergoes validation [43]. Gholamiangonabadi et al [44] demonstrated that LOSOCV serves as a rigid criterion for evaluation models of times series accelerometer data in human activity recognition.

In this study, the process involved 6 iterations and, for each iteration, data from 1 out of the 6 participants was left out as the validation set to train the XGBoost classification model.

### Ethical Considerations

Ethical approval to conduct the research was obtained from the KU Leuven Social and Societal Ethics Committee with protocol number G-2020‐2214. All participants were invited to participate voluntarily and received verbal and written information about the study in advance. Each participant signed an informed consent form and was assigned a unique identifier for data processing. The first author kept the names and unique identifiers separately from the obtained study data.

## Results

### Algorithm Performance

Upon acquisition of data from all participants, the data were processed to assess the effectiveness of the XGBoost model for the task at hand. The validation outcomes are represented in Tables2  3, with the results for the total dataset from two Byteflies dots at the top and the results split per Byteflies dot at the bottom. In total, the dataset encompasses 898 windows, of which 481 windows (53.56%) were attributed to the class “In bed,” 60 windows (6.68%) to “Agitation,” 207 windows (23.05%) to “Turn,” and 150 windows (16.70%) to “Out of bed.” Because the number of windows per class is not proportional, the dataset can be considered imbalanced.

**Table 2.** Overview of the distribution of windows (n and %) per class for the total dataset from two Byteflies dots and the classification metrics precision, recall, and *F*_1_-score (%) per class for the leave one subject out cross-validation of the XGBoost model.

	In bed	Agitation	Turn	Out of bed
Windows, n (%)	481 (53.6)	60 (6.7)	207 (23)	150 (16.7)
Precision (%)	84.32	77.78	69.67	78.95
Recall (%)	86.07	81.67	71.01	70
*F*_1_-score (%)	85.19	79.67	70.33	74.24

**Table 3.** Overview of the classification metrics precision, recall, and F1-score (%) per Byteflies dot (d1 and d2) for the leave one subject out cross-validation of the XGBoost model.

	In bed		Agitation		Turn		Out of bed	
	d1	d2	d1	d2	d1	d2	d1	d2
Precision (%)	81.75	77.99	84.13	68.06	71.83	59.46	77.97	80.70
Recall (%)	85.65	85.45	88.33	81.67	73.56	52.88	61.74	61.74
F_1_-score (%)	83.65	81.55	86.18	74.24	72.68	55.98	68.91	69.96

**Figure 4.**
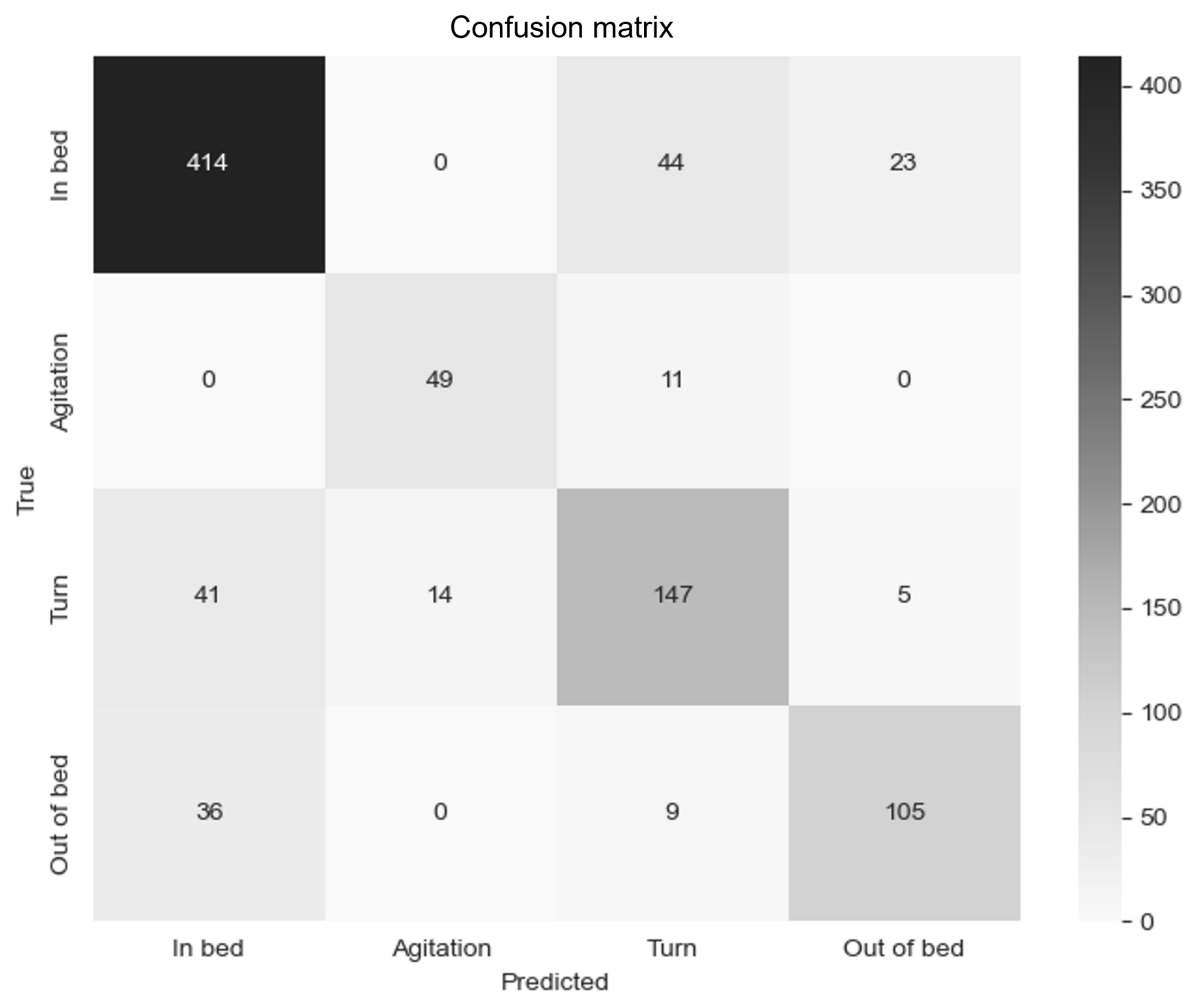
The confusion matrix of the leave one subject out cross-validation of the XGBoost model, containing data from 2 Byteflies dots, visualizing the number of windows predicted per class.

The classification metrics (ie, precision, recall, and *F*_1_-score) listed in Tables2  3 provide a performance assessment of the deployed algorithm. The confusion matrix depicted in Figure 4 illustrates the distribution of the number of windows predicted per class for the total cross-validation dataset from two Byteflies dots. Precision is the measure to tell how many correct positive predictions the model made [26]. It is calculated as the ratio of true positive predictions to the total positive predictions (true and false positive). The class “In bed” attained the highest precision (84.32%) and the model interpreted a few of the windows from the classes “Turn” (41) and “Out of bed” (36) as “In bed.” These are called false positives. The precision for “Agitation” was 77.78% as the model predicted some windows from the class “Turn” as “Agitation” too.

Recall is another classification metric to measure the ratio of correct positive predictions to all actual positives [26]. The recall for the class “In bed” scored the highest (86.07%), and the misclassified windows were predicted as “Turn” (44) or “Out of bed” (23) (false negatives). Also for the “Agitation” class, it was observed that 11 of the incorrectly predicted windows were labeled as “Turn.” Conversely, the incorrect predictions for “Turn” also had a majority of 41 windows in “In bed.” For the class “Out of bed,” the most incorrectly predicted windows were observed in “In bed” (36).

At last, the *F*_1_-score is the harmonic mean or weighted average of precision and recall for a classification problem and especially useful with an imbalanced dataset [26]. The class “In bed” attained the highest overall *F*_1_-score (85.19%) and “Turn” manifested the lowest *F*_1_-score (70.33%).

The results split per Byteflies dot in Table 2, revealed an overall better outcome for dot 1, compared with dot 2, especially for the classes “Agitation” and “Turn.” When combining the data from two dots, the results for the classes “In Bed” and “Out of bed,” improved. In contrast, the results for “Agitation” and “Turn” were higher for dot 1 than combined with the lower result of dot 2.

### High-Impact Features

**Figure 5.**
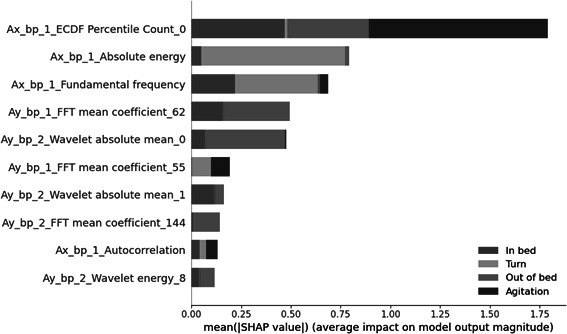
The bar plot of the mean absolute Shapley additive explanations values per class for the top 10 features with the highest impact on the XGBoost model’s output. The name of the feature provides information on the data used to compute the feature: the acceleration axis (Ax, Ay, or Az), the applied band-pass filter (bp), and the sensor dot (1 or 2). ECDF: Empirical Cumulative Distribution Function; FFT : Fast Fourier Transform.

Figure 5 illustrates the mean absolute SHAP value per class for the top 10 features with the highest impact on the model predictions. SHAP values use a game-theoretic approach to quantify the contribution of each feature to the machine learning model’s outcome [45]. These values assign an importance value to each feature, reflecting how much it influences the final prediction. To specifically elaborate on the tree-based XGBoost model at hand, the TreeExplainer explanation method is used. The name of the feature provides information on the data used to compute the feature: the axis (Ax, Ay, or Az), the applied filter (band-pass), and the sensor dot (1 or 2).

Among the selected features, the “ECDF Percentile Count” computes the cumulative sum of samples falling below the percentile of the empirical cumulative distribution function (ECDF) [37].

The ECDF is a simple nonparametric estimator and is obtained by calculating the cumulative probability for each number of unique observations in the data sample less than or equal to a given unique observation x, divided by the total number of observations n (Equation 1) [46,47].


(1) $$ECDF(x)=(number\;of\;unique\;observations\leq \frac{x}{n})$$

Notably, the “ECDF Percentile Count_0” for data along the x-axis of sensor dot 1 computes the cumulative sum of samples falling below the 20th ECDF percentile and significantly impacts the model’s output across the three classes “In bed,” “Out of bed,” and “Agitation.” For the class “Turn,” the feature “Absolute energy” for data of the x-axis of sensor dot 1, which computes the absolute energy of the signal, has the highest average 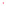
impact [37]. In addition, 3 features in the plot involve FFT mean coefficients, capturing the mean value of each spectrogram frequency [37]. With a default setting of 256 bins and a sampling frequency of 100 Hz, the bin width is calculated with Equation 2 and is 0.39 Hz. This means that bin 55 corresponds to 21.48 Hz, bin 62 to 24.22 Hz, and bin 144 to 43.68 Hz. The frequency of bin 144 is the same as for bin 112, as only half of the bins are unique in the FFT spectrum of a signal [48].


(2) $$Bin\;width=\frac{Sampling\;frequency}{Number\;of\;bins}=\;\frac{100\;Hz}{265\;bins}=0.39\;Hz$$

Another set of 3 features in the plot relates to continuous wavelet transform (CWT): “Wavelet absolute mean”’ computes the CWT absolute mean value for each wavelet scale, while “Wavelet energy” quantifies the CWT energy for each wavelet scale [37]. Importantly, none of the selected features are computed based on z-axis data. Based on the positioning of the sensor dots, the z-axis was directed upwards, from the bottom to the top of the mattress [32].

### Leave One Subject Out Cross-Validation

Table 4 presents the weighted F1-score and accuracy for the LOSOCV set per participant, along with the overall result. The accuracy, or the ratio of correct predictions to the total number of predictions [26], is chosen to be able to compare the results with prior work. However, the weighted F1-score is the more appropriate metric here for model validation due to the label imbalance of the dataset, as illustrated in Table 2 and Table 3. The model achieved an overall score of 79.56% for F1-score and 79.62% for accuracy. Participant-specific performance ranged from a minimum F1-score of 69.40% (participant 3) to a maximum of 87.72% (participant 4), and accuracy ranged from 69.33% (participant 3) to 88% (participant 4).

**Table 4.** Overview of the weighted *F*_1_-score and accuracy (%) per participant (p) and in total for the leave one subject out cross-validation of the XGBoost model, containing data from two Byteflies dots.

	p1	p2	p3	p4	p5	p6	Total
*F*_1_-score (%)	80.71	79.27	69.40	87.72	77.37	81.67	79.56
Accuracy (%)	80.54	79.33	69.33	88.00	78.00	82.55	79.62

## Discussion

### Principal Results

The study gained insights into using accelerometer sensors, an XGBoost model, and LOSOCV as an unobtrusive approach for monitoring nighttime movements to support NH continence care, using a viscoelastic care mattress in our setup, which effectively distributes an individual’s weight over a broader surface area.

The confusion matrix indicated that the model correctly classified most windows. With an overall *F*_1_-score of 79.56%, and more specifically 79.67% for the class “Agitation,” the algorithm developed in this study has attained a high level of trustworthiness. The validation results for each participant revealed a variation in *F*_1_-score of 18.32% among participants. Despite this variability, it is noteworthy that all participants’ test outcomes achieved strong model performance. This was particularly remarkable given the considerable differences in weight and height among participants.

A notable observation is applied to the results for class “Turn,” where 41 windows are misclassified as “In bed.” This misclassification may be attributed to the selected window size of 20 seconds. This window size provided the best overall result during the exploration phase, but is considerably larger than the 10-second duration of a turn in the dataset. Given that the primary emphasis of the study was on the detection of agitation, this misclassification was not deemed as concerning. Another issue arose with the smaller number of misclassifications between “In bed” and “Out of bed.” By removing the signal’s direct current component with a band-pass filter, the difference between the 2 activities became less visible. Given the importance of being able to accurately determine whether the bed of an NH resident is occupied or not, the sensor system could be enhanced by incorporating additional components, such as a pressure sensor.

In our investigation of feature impact on the output model, the cumulative sum of samples falling below the 20th percentile of the ECDF has a high impact on 3 of the 4 classes: “In bed,” “Out of bed,” and “Agitation.” Interestingly, none of the selected features are computed based on the z-axis data. This suggests that movement in the (inverse) direction from the bottom to the top of the mattress provides less informative input for our classification task.

Upon comparing the outcomes for the individual sensor dots with the combined result, it became evident that using more than one sensor only slightly improved the model’s overall performance for 2 out of 4 classes. When learning more about the results per dot for “Agitation,” it was observed that only 4 additional windows were misclassified for dot 2, compared with the result for dot 1. Since there was only a limited number of windows for this class, this is immediately notable in a performance assessment. Unfortunately, there was no clear explanation for this discrepancy in the classification.

In practical application, a nighttime movement monitoring system could support (continence) care in NHs by notifying care personnel based on the detected events. In the case of the detected event “In bed,” no immediate action from care personnel is required. However, when the system detects “Agitation,” care personnel should receive a notification, especially when multiple successive agitation events are identified. For incontinent residents, care personnel could then assess and, if necessary, change the incontinence material. To provide personalized support, the NH would have the flexibility to adjust the threshold for the number of detected events per resident. For the event “Turn,” action from care personnel would only be necessary when it occurs infrequently, aimed at preventing decubitus. Here, the frequency could be tailored to each resident. Finally, when “Out of bed” is detected, care personnel should be notified that the resident has left the bed, allowing them to assist the resident back into bed without further complications.

### Comparison With Prior Work

Gong et al [19] detected agitation using data from both bed sensors and wristbands for the algorithm, yet the paper lacks detailed precision insights or evaluation metrics for their model to compare with the results of this study.

T’Jonck et al [20,21] achieved a high accuracy (92% and 85%) using FFT and CNN models across various bed sensor setups and participants. Their studies, however, entailed fewer participants and did not use a viscoelastic care mattress.

Notably, the smartphone accelerometer placed on top of the mattress yielded higher accuracy than the bottom placement of the accelerometer sensor, indicating the latter as a more challenging position for movement measurement. Nonetheless, the bottom placement aligns more closely with the goal of developing an unobtrusive system. T’Jonck et al [20] also concluded that the accelerometer’s position on the bed should not significantly impact the model’s ability to classify the data. This is contradictory to our findings, where a difference in performance is recorded between the two Byteflies dots.

### Limitations

The dataset of our study is limited, incorporating only 6 iterations (1 per participant). A larger sample size by including more participants in the study could potentially yield improved results.

Another limitation was the absence of NH residents or older adults among the participants. The simulation of large body movements was based on participants’ own interpretation, which may not authentically mirror the nocturnal agitation experienced by older adults.

Finally, the study adhered to a protocol designed to simulate nighttime movement. It is essential to note that this simulation differs from real nighttime movement, where lying in a specific posture, turning, or experiencing agitation is not dictated by predefined time intervals and does not necessarily follow a sequential pattern.

### Conclusions

This study presented the exploration of accelerometer-based unobtrusive monitoring of nighttime movements to support NH continence care. The XGBoost model combined with an LOSOCV approach provided valuable insights into activity tracking. The model was able to successfully detect the specified activities with an overall *F*_1_-score of 79.56%.

To gain deeper insights into the developed sensor system and to address its limitations, we recommend conducting a follow-up study in an NH setting. This will enhance the study’s external validity by capturing real-world conditions. Incorporating NH residents and monitoring their nighttime behavior present new challenges, including limited bed mobility and the need for transfers.

## Abbreviations

CNN: convolutional neural network
CWT: continuous wavelet transform
ECDF: empirical cumulative distribution function
FFT: fast Fourier transform
LOSOCV: leave one subject out cross-validation
NH: nursing home
SHAP: Shapley additive explanations
UI: urinary incontinence
